# Unpaid Medical Expenses by Foreign Patients in Japan: A Scoping Review

**DOI:** 10.31662/jmaj.2024-0418

**Published:** 2025-12-26

**Authors:** Soichiro Saeki, Hatsune Kido, Chihaya Hinohara, Yan Zhang, Eri Yamada, Kaori Minamitani, Ryo Kawasaki

**Affiliations:** 1 Division of Public Health, Department of Social Medicine, Graduate School of Medicine, Osaka University, Suita, Japan; 2 Department of Emergency Medicine and Critical Care, Center Hospital of the National Center for Global Health and Medicine, Tokyo, Japan; 3 Department of Medical Education, Kurashiki Central Hospital, Kurashiki, Japan; 4 Multicultural Communication Support Team, Kurashiki Central Hospital, Kurashiki, Japan; 5 International Health Care Center, Center Hospital of the National Center for Global Health and Medicine, Tokyo, Japan; 6 Department of International Medical Care, Rinku General Medical Center, Izumisano, Japan

**Keywords:** migrant health, minority health, travel insurance, payment, foreigners, universal health coverage

## Abstract

**Background:**

The increase in international tourists and residents in Japan has necessitated Japanese medical institutions to extend their services to patients, presenting new challenges such as unpaid medical bills. The treatment of foreign patients is further complicated by linguistic, cultural, and social barriers, which heighten the risk of unpaid bills. This highlights the critical need to examine the issue of unpaid medical bills in the context of foreign patient care in Japan. This study investigates trends in research, the regions most affected, and the impact of unpaid medical expenses on patients and healthcare institutions.

**Methods:**

A scoping review was conducted using articles indexed in MEDLINE, Web of Science, Scopus, CINAHL Plus, Ichushi-Web (Japanese medical literature), and Google Scholar, with a focus on publications from Japan discussing unpaid medical bills with foreign patients.

**Results:**

Seventeen publications met the criteria, highlighting the difficulties in collecting medical expenses from foreign patients. Prevention methods, such as prepayment and presenting estimated costs before care were suggested but had limited success. There are also few policies to assist medical institutions with outstanding expenses from foreign patients.

**Conclusions:**

This study underscores the urgent need for comprehensive strategies to address unpaid medical bills among foreign patients in Japan, emphasizing further research to develop effective interventions and enhance the overall healthcare experience for these patients.

## Introduction

With the advancement of globalization, the number of individuals traveling or residing abroad has increased ^(1)^. Japan is no exception ^(2)^, as the influx of international tourists visiting the country has increased approximately sixfold since the early 21st century until 2019 ^(3)^. With respect to foreign nationals residing in Japan, 2.20% of the nation’s population comprised non-Japanese residents in 2021 ^(3)^.

Numerous issues concerning healthcare services provided to foreign patients in Japan have been documented ^(4), (5), (6), (7), (8), (9), (10), (11), (12), (13)^. This encompasses medical care for new immigrants or their descendants, who may possess medical histories distinct from those of local patients ^(9), (14), (15)^, as well as healthcare for international travelers grappling with various health concerns far from their native countries ^(8), (16), (17), (18)^.

However, the healthcare needs of non-Japanese patients have historically been underappreciated in Japan ^(19), (20)^. Measures have been implemented to address the rise in international tourists, including practical guidance for patients with limited Japanese proficiency ^(21)^ and accrediting medical facilities for foreign patients. These efforts accelerated in preparation for the Tokyo Olympics, but many hospitals remain ill-prepared ^(22)^. A national survey indicated that 95% of Japanese hospitals worry about linguistic issues with foreign patients ^(23)^, but further research into foreign patient care is lacking ^(24)^.

One principal concern when providing healthcare for foreign patients is the payment of medical expenses ^(25)^. The Japanese National Health Insurance system offers a universal health coverage system that covers healthcare costs that allow both Japanese citizens and foreign residents to join and receive the necessary insurance benefits in the event of illness or injury ^(26)^, limiting patients’ copayment to 10%-30% (0% for patients under welfare) ^(27)^. A system also caps the maximum healthcare expenses for hospitalization, depending on the previous year’s income ^(28)^. Foreign nationals with valid residency visas are eligible for this public healthcare insurance, provided that they pay insurance premiums. Hence, the system relies on trust in subsequent payments. Payments for outpatients are usually made after consultations and procedures, and for inpatients, the final expense is calculated and billed on the day of discharge. Furthermore, doctors are obliged to treat patients in Japan, and regardless of their nationality or public medical insurance, they provide necessary medical services.

However, foreign residents not enrolled in the national healthcare system, such as asylum seekers or those with expired visas ^(29)^, and travelers from overseas are excluded from this coverage, rendering these individuals more vulnerable to substantial medical expenses. In addition to these groups, structural vulnerabilities exist even among long-term foreign residents. Although Japan’s social health insurance system ensures universal coverage in principle, it is restricted to individuals with legal residency of at least 3 months ^(30)^. Consequently, short-term residents and undocumented migrants are excluded. Moreover, even eligible foreign residents may become disenrolled from National Health Insurance due to non-payment of premiums or loss of employment-based coverage. Lack of knowledge of enrollment requirements, combined with language and administrative barriers, can contribute to unintentional loss of insurance coverage ^(30)^. In 2025, Shinjuku City highlighted low National Health Insurance premium payment rates among foreign residents ^(31)^. These systemic factors may result in foreign residents facing full out-of-pocket medical costs, further increasing the risk of unpaid medical bills.

Additionally, the treatment of foreign patients is further complicated by linguistic, cultural, and social barriers, which increase the risk of unpaid bills. Owing to the differences in medical systems between countries, particularly in systems for paying for treatment, there is a problem of uncollected payments, where foreigners do not pay the medical fees stipulated by the Japanese medical system, and medical institutions cannot receive payments for treatment. Consequently, healthcare providers and medical institutions have expressed concerns about the medical costs of foreign patients in Japan, especially since these traveler patients may not stay long-term, making cost recovery more challenging than for residents.

Despite this, limited evidence has been gathered regarding Japan’s current situation, preventive measures, and risk reduction strategies for unpaid medical expenses. To ensure the sustainability of Japan’s medical insurance system, it is necessary to understand and organize the issues surrounding uncollected payments for medical treatment for foreign residents. Thus, we conducted a scoping review of studies within Japan to offer healthcare providers, medical facilities, and potential patients’ insights into the current landscape and possible preventive strategies concerning unpaid medical expenses by foreign patients in Japan.

## Materials and Methods

We performed a scoping review concentrating on foreign patients in Japanese emergency departments. This review was conducted following the PRISMA extension for scoping reviews (PRISMA-ScR) guidelines ^(32)^.

### Search strategy

Six databases, PubMed, Web of Science, Ichushi-Web (Japanese medical literature), Scopus, CINAHL Plus, and Google Scholar, were initially searched on July 9, 2024, to create an outline of the review. The same databases were researched for the formal search on October 10, 2024. The search keywords used were “Japan,” “foreign patients,” “unpaid medical bills,” “outstanding expenses,” and “payment.” The search was limited to English- and Japanese-language manuscripts conducted in Japan. The duration and type of study or publication were not limited. The search strategy is provided in Table 1. In addition, a search for gray literature was conducted by the First Author on July 9, 2025, using two databases: Google Scholar and Ichushi-web, using the same terms in Japanese. Although some municipal governments, such as Shinjuku City have highlighted low National Health Insurance premium payment rates among foreign residents, which may indirectly contribute to the occurrence of unpaid medical bills at hospitals and clinics ^(31)^, as these reports do not directly address unpaid bills at medical institutions, they were not included in our formal dataset.

**Table 1.** Search Strategy.

Item	Details
Database	PubMed, Web of Science, Ichushi-web (Japanese medical literature), Scopus, CINAHL plus
Other sources	None
Key searched terms	“Japan”, “foreign patients”, “unpaid medical bills,” “outstanding expenses,” “payment”
Language	English, Japanese
Location	Japan
Duration	Not limited
Type of Study	Not limited
Type of Publication	Not limited

The search strategy of the scoping review is presented.

### Eligibility criteria

All study designs that were (1) focused on foreign patients, (2) conducted in or reported from Japan, and (3) reported any comments regarding unpaid medical expenses by foreign patients were included in this study review. The review also included case reports and gray literature to gain an in-depth view of why unpaid medical expenses occurred.

### Study selection and data extraction

For the formal search, two authors independently reviewed every title and abstract of the identified manuscripts on the basis of the eligibility criteria, wholly blinded by each author, via the Rayyan platform ^(33)^. The full-text papers were read to confirm the final inclusion decision. Disagreements were resolved upon discussion with the authors.

One author independently retrieved the data from the full-text articles presented to report for this study. For case reports, the following information was extracted: publication year, nationality of the patient discussed in the case, visa (residency) type of the patient, diagnosis of the case, treatment conducted through the case, outcome, unpaid medical bills that occurred from the case, and discussions by the authors regarding unpaid medical bills. The following information was extracted from the original studies: publication year, study duration, study type, location, study population, the total number of patients and facilities included in the study, total unpaid medical expenses, the total number of cases discussed in the study, key findings, and discussions on unpaid medical bills. No meta-analysis was conducted due to the nature of this study.

### Data charting

Data charting was conducted to visualize the abstracts of the studies included in the review. A word cloud was created to assess the main topics of the study. The full text of each manuscript was analyzed via Google Collaboratory (Google LLC, Mountain View, CA, USA) to create a word cloud. Maps and graphs were created via Microsoft Excel (Microsoft Corporation, Redmond, WA, USA). The maps’ credentials were automatically made in Microsoft Excel and are shown in the graphics, as designated by their print rights ^(34)^.

## Results

### Study inclusion criteria

**Figure 1.**
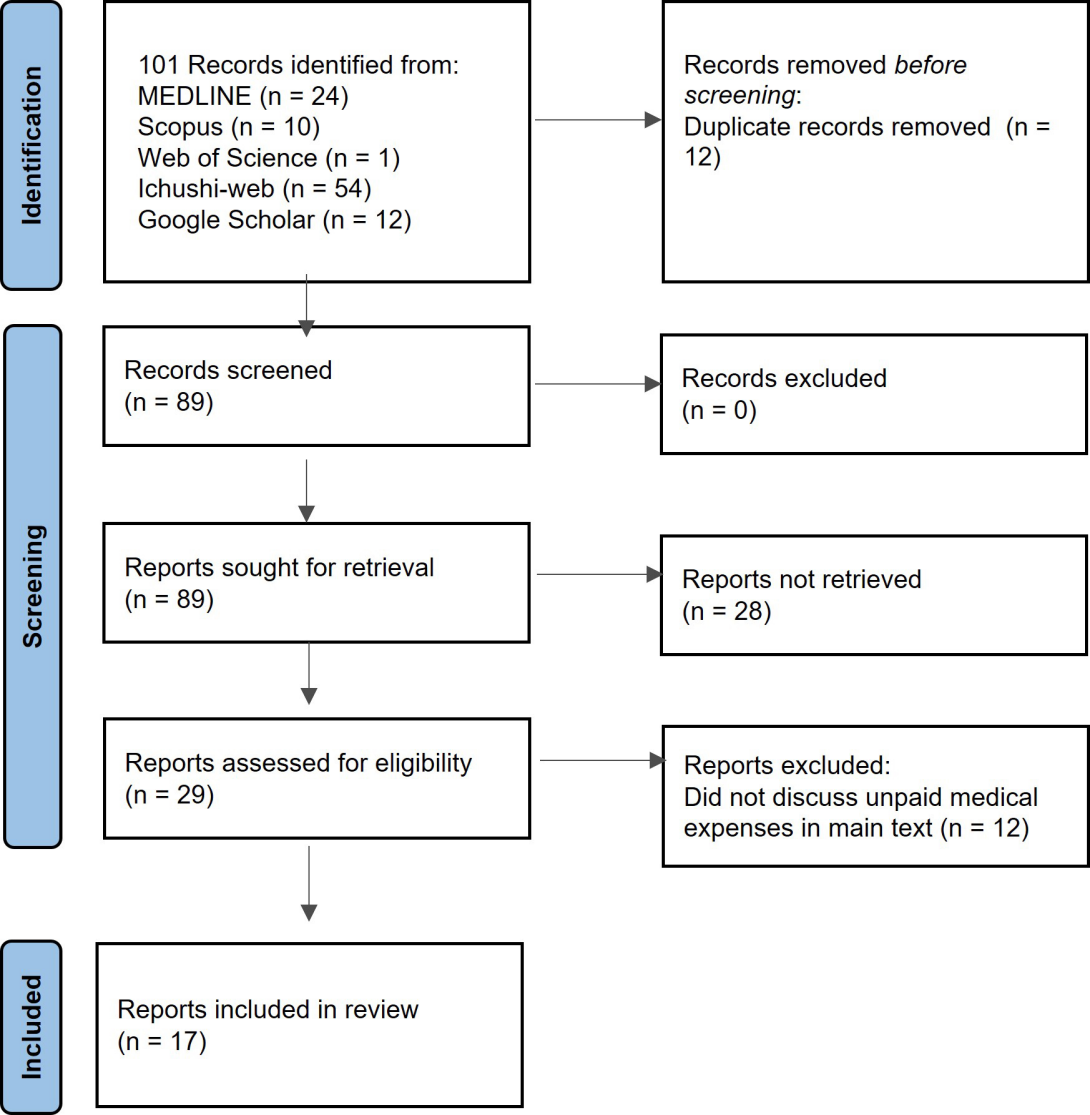
Inclusion and exclusion criteria for the studies included in the study. The study was conducted following the PRISMA-ScR guidelines.

Figure 1 shows the inclusion and exclusion process according to the PRISMA-ScR statement. Overall, 101 references were obtained after the database was searched, and 12 were identified as duplicates. Upon initial title and abstract screening, 29 papers satisfied the inclusion criteria. Following a full-text review, 12 studies were excluded because they only discussed payment issues among foreign patients but did not comment on unpaid medical bills or prevention strategies. Finally, 17 manuscripts were evaluated qualitatively.

#### Description of the included studies

**Figure 2.**
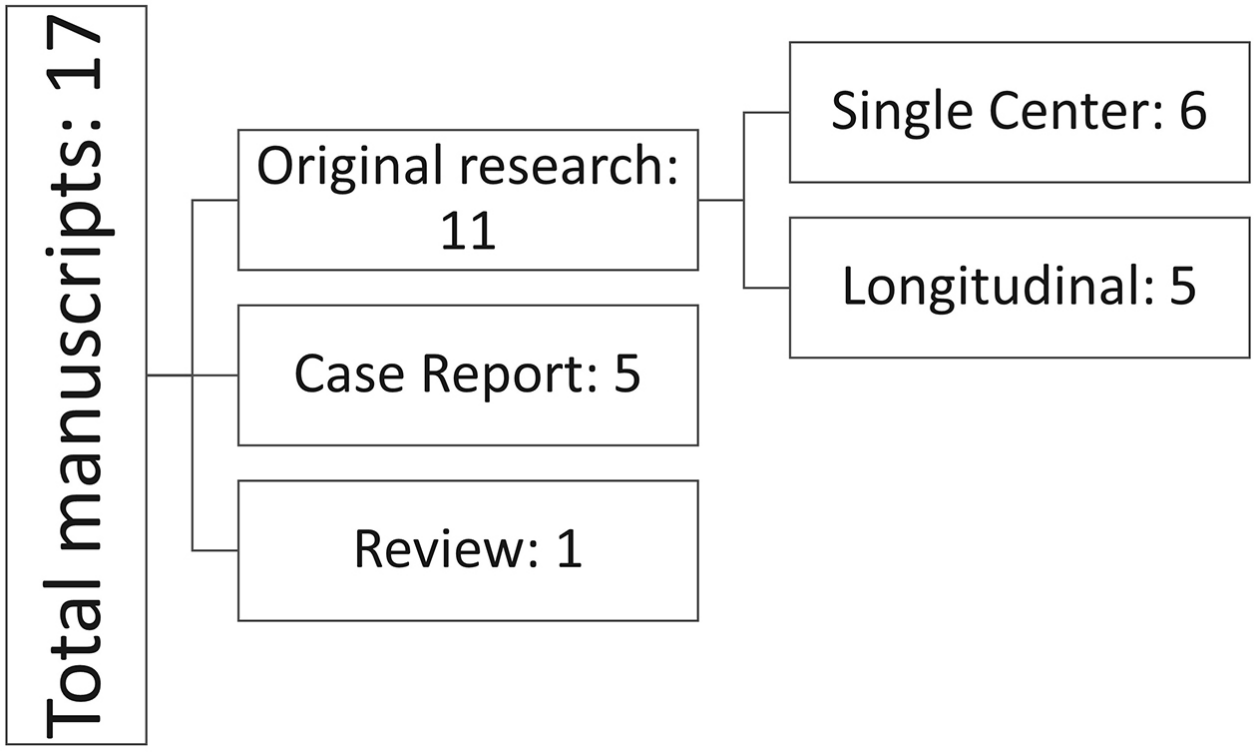
Outline of the studies included in the review. The key findings of each study are described in Table 2.

The outlines of the articles are presented in Figure 2. Among the 17 studies identified, five ^(35), (36), (37), (38), (39)^ were case reports, 11 were original studies, and one was a review article. Among the 11 original research articles, five studies ^(40), (41), (42), (43), (44)^ were longitudinal studies that conducted questionnaires or interviews with medical facilities, healthcare providers, or patients, and six studies ^(45), (46), (47), (48), (49), (50)^ were single-center, retrospective reviews of cases in hospitals. The review article ^(51)^ was a systematic review focused on foreign patients visiting emergency departments in Japan, and studies identified in that review regarding unpaid medical bills among foreign patients were also included.

In addition to peer-reviewed studies, various gray literature sources―such as institutional case reports and conference proceedings―have documented the increasing burden of unpaid medical expenses involving foreign patients in Japan. The list of these sources is provided as Supplementary Material. These sources highlight practical countermeasures, such as multilingual billing forms, pre-admission payment protocols, the introduction of credit card payment options, and the establishment of dedicated billing departments.

The geographic features of the studies are shown in Figure 3. Figure 3a shows the prefectures of the case reports, and Figure 3b shows the prefectures of the original studies. The locations are concentrated mainly in areas where the population is concentrated in Japan. Regarding the years of publication, two peaks were observed at the end of the 20^th^ century and after 2010 (Figure 4).

**Figure 3-a.**
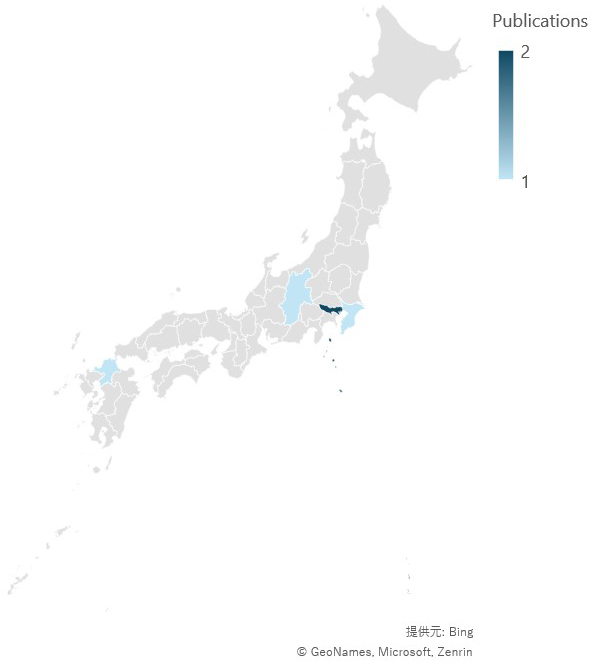
Locations of the case reports included in the review. Maps were created via Microsoft Excel. The credentials are presented as is.

**Figure 3-b.**
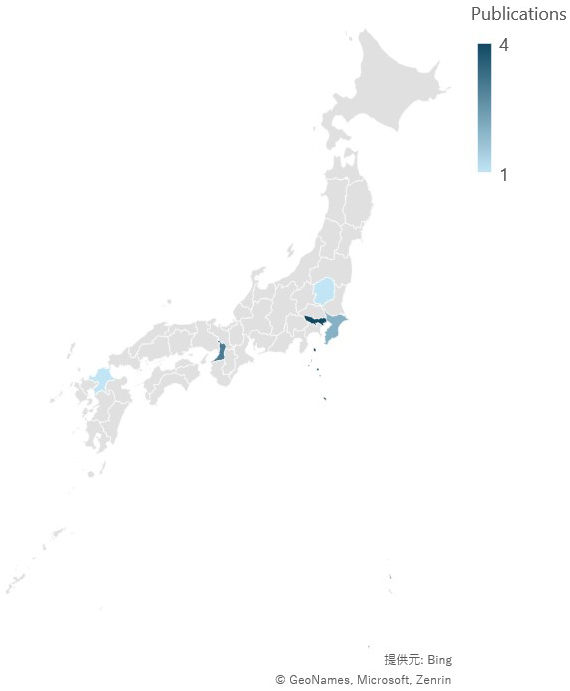
Locations of the original studies included in the review. Maps were created via Microsoft Excel. The credentials are presented as is.

**Figure 4.**
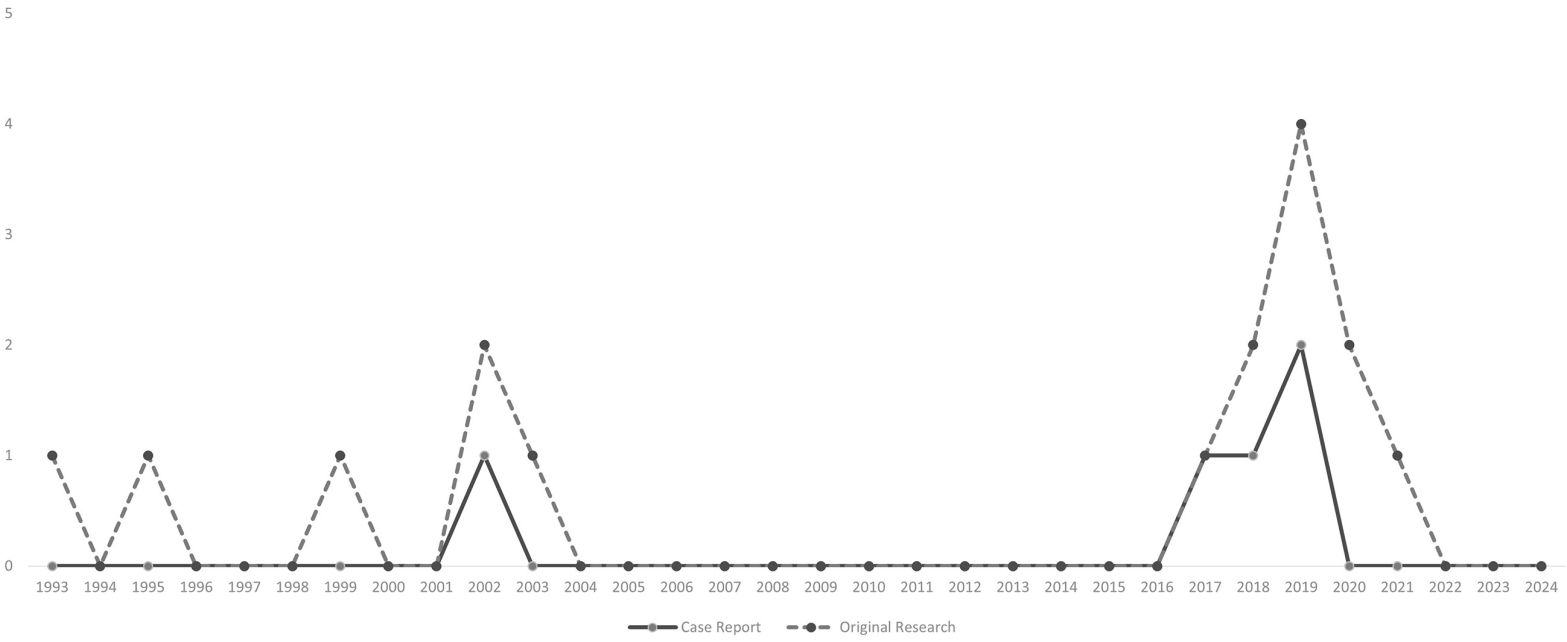
The number of studies published per year. Case reports and original research are presented. The review article was published in 2023.

**Figure 5.**
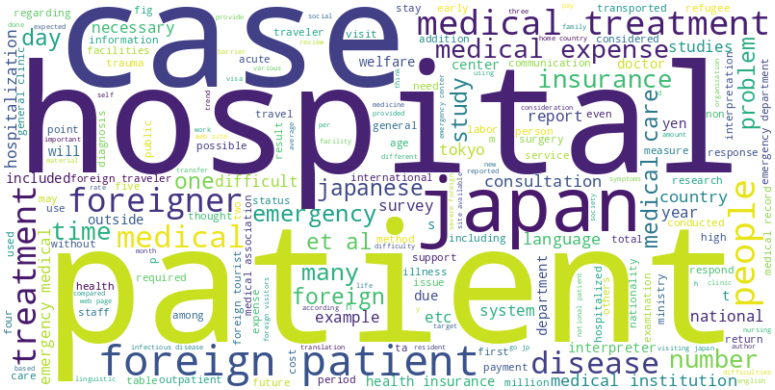
Word cloud of the studies in the manuscripts included. The full texts of the studies included were analyzed.

The word cloud of the full texts is presented in Figure 5. The contents highlighted in the word cloud are not necessarily directly related to unpaid medical bills, suggesting that the studies included in the review did not focus mainly on unpaid medical bills but focused on other topics related to outstanding expenses.

### Overview of case reports

Table 2a shows an overview of the five case reports that included a discussion of unpaid medical expenses by foreign patients in Japan. One study ^(35)^ reported two cases, and the total number of cases presented was six. Four cases involved temporary visitors (tourist visas), which did not qualify patients for public health insurance in Japan. The diagnosis ranged from acute to chronic, and in addition to the two studies ^(38), (39)^ that did not mention the amount of unpaid medical expenses, all the cases presented unpaid medical expenses. Discussions regarding outstanding expenses included prepayment or presenting estimated medical expenses beforehand to prevent unpaid medical expenses. They commented on discussions among other parties, such as the lack of legislative subsidies to aid medical facilities or difficulty communicating with companies and nonprofit organizations that support patients.

**Table 2-a.** Overview of Case Reports Presenting Unpaid Medical Bills by Foreign Patients.

#	Author	Year	Age	Nationality	Visa type	Diagnosis	Treatment	Outcome	Unpaid medical bills	Discussions on unpaid medical bills
1-1	Akasaka et al. ^(35)^	2002	27	Philippines	Unspecified	Kidney failure	Immediate hemodialysis upon hospitalization	Recovering	430,000 JPY	Although the company that supported the patients’ coming to Japan was to pay the bills, the payment was never made.
1-2	Akasaka et al. ^(35)^	2002	27	Philippines	Overstay (formerly spouse or child of Japanese National)	Kidney failure	Hemodialysis, lithotripsy	Transfer	2,500,000 JPY	There was no financial support from the public sector as the patient’s visa had expired.
2	Shoko et al. ^(36)^	2017	34	Asian (unspecified)	Temporary	Fulminant hepatitis B, hepatic failure	Antiviral drugs, blood transfusion, plasma exchange, antibiotics	Death	Fully paid by relatives residing in Japan (approximately 6,000,000 JPY)	Preparation before the patient’s visit, mainly presenting the estimated medical bills, is critical. Although several public aids are available for medical facilities with unpaid medical bills by foreign patients, the conditions for application are strict.
3	Fujita et al. ^(37)^	2018	50	Palestine (Refugee)	Temporary (later changed to Designated Activities)	Type 2 diabetes mellitus, chronic kidney disease, cellulitis, diabetic gangrene, hepatic cirrhosis	Hemodialysis, antibiotics, wound care	Recovering	455,371 JPY	The patient could not obtain Japanese healthcare insurance as the residency visa was not obtained until 1 month after admission. Using the free/low-cost medical service for refugees may be an option.
4	Akashi et al. ^(38)^	2019	30s	China	Temporary	Breast cancer stage IIB T2N2M0	Biopsy, preoperative chemotherapy, surgery, radiation therapy	Recovering	N/A	Preparation before the patient’s visit, mainly presenting the estimated medical bills, is critical.
5	Matsumoto et al. ^(39)^	2019	40s	Southeast Asian (unspecified)	Temporary (visiting relatives in Japan)	Acute cerebral infarction (branch atheromatous disease)	Immediate antithrombotic therapy. Antiplatelet therapy using generic drugs. Rehabilitation therapy by family members after early discharge.	Recovering	N/A	Several public aids are available for medical facilities with unpaid medical bills by foreign patients. Legislative measures for infectious diseases and systems such as workers’ compensation insurance and mandatory vehicle liability insurance may be usable under specific circumstances. Prepayment may also be an option to prevent unpaid medical bills.

The year of publication, patient nationality, visa type, diagnosis, treatment, outcome, unpaid medical bills, and discussions on unpaid medical bills were derived from each study. JPY, Japanese yen; N/A, not available.

### Overview of longitudinal studies

The longitudinal studies that described unpaid medical expenses by foreign patients in Japanese medical facilities are summarized in Table 2b. Three studies dated back to the 20^th^ century ^(40), (41), (42)^, whereas two were conducted in 2018 ^(43), (44)^. All the studies reported multiple cases of unpaid medical bills by foreign patients. For example, Shima et al. ^(42)^ reported 23.8 million yen in unpaid bills in a 1-year period in medical facilities across Chiba City in a total of 20 medical facilities, and Ishikawa et al. ^(46)^ noted a non-payment rate of approximately 20% among all foreign patients at a tertiary hospital in Tokyo across a 7-year period. One study included qualitative research with interviews with overseas students.

**Table 2-b.** Overview of Longitudinal Studies Presenting Unpaid Medical Bills by Foreign Patients.

#	Author	Year	Duration	Study type	Location	Population	Total patients/facilities	Total Unpaid Medical Expenses and cases	Key findings	Discussions on unpaid medical bills
6	Kunii et al. ^(40)^	1993	May-July 1991	Questionnaire among medical facilities in Tochigi prefecture	Tochigi	1216 medical facilities in Tochigi prefecture	558 medical facilities	4,476,196 JPY (max 3,275,830 JPY)	Two-thirds of foreign patients did not have any health insurance. Foreign patients were reluctant to visit healthcare facilities due to communication and financial issues.	Hospitals may be reluctant to accept foreign patients due to the risk of large outstanding expenses. Discussions to encourage foreigners to obtain healthcare insurance are necessary.
7	Momose and Esaki ^(41)^	1995	June 1993 (Survey) August 1993 (Interview)	Questionnaire, Interview	Fukuoka	Questionnaire towards physicians in medical facilities that were listed as “medical facilities with physicians speaking foreign languages”; interviews toward university exchange students	253 physicians (survey), 37 exchange students (interview)	16 (12 in clinics, 4 in hospitals, both amounts unspecified).	Communication was difficult due to linguistic barriers. Patients had difficulty accessing healthcare information from the public sector. 6.3% of physicians experienced unpaid medical bills, and only insufficient treatment was available to some patients with a lack of ability to pay medical expenses.	Unpaid medical bills were observed more commonly in clinics (12%) than hospitals (2.6%). Clinics claimed the bills to the employers of the patients.
8	Shima et al. ^(42)^	1999	October, 1997	Longitudinal questionnaire study	Chiba	467 medical facilities in Chiba city	210 medical facilities	20 facilities, a total of 23,800,000 JPY over the past year.	133 facilities (63.3%) had foreign patients. The main problems encountered with foreign patients were linguistics and payment for those without national medical insurance.	Some facilities use insurance that covers unpaid medical bills by foreign patients. Policies should be made to aid foreign patients in obtaining insurance and reimbursements for unpaid bills. Information on health insurance should be disseminated to foreigners who are eligible for public health insurance.
9	Kishi et al. ^(43)^	2018	January-July 2018	Longitudinal questionnaire study	Osaka	179 medical facilities with emergency departments in Osaka prefecture	78 medical facilities	28 facilities (38%) experienced unpaid medical bills with foreigners.	71% of the facilities had difficulty as they had no interpreters.	Reasons for unpaid medical bills were lack of cash, not having travel insurance, and difficulty receiving payment after returning to the homeland.
10	Kishi et al. ^(44)^	2020	January-December 2018	Longitudinal questionnaire study	Osaka	282 medical facilities with emergency departments in Osaka prefecture	145 medical facilities	53 facilities (56%) experienced unpaid medical bills with foreigners.	72% of the facilities had difficulty as they had no interpreter. Issues regarding patients’ perception of medicine, religion, diet, and patient transfers arose as new problems in hospitals.	Although Osaka prefecture organized a consultation desk regarding foreign patient care, they can only provide essential information and do not provide reimbursement.

The year of publication, duration and location of the study, study population, total number of patients or facilities included in the study, total amount of unpaid medical expenses reported, key findings, and discussions on unpaid medical bills were derived from each study. JPY, Japanese yen.

While the first three studies focused mainly on healthcare coverage for foreign residents in Japan ^(40), (41), (42)^, the latter two focused on foreign tourists’ outstanding medical expenses ^(43), (44)^. The former ^(40), (41), (42)^ discussed expanding public health insurance coverage toward foreigners, whereas the latter ^(43), (44)^ discussed problems such as travel insurance. Both studies noted the lack of policies, such as reimbursements and other legislative measures to support medical facilities that treat foreign patients.

### Overview of retrospective studies

Retrospective studies that described unpaid medical expenses by foreign patients in Japanese medical facilities are summarized in Table 2c. All analyses were conducted through a retrospective review of healthcare records in a single medical center. Most studies targeted foreign patients visiting the emergency department, and these studies reported unpaid medical bills from foreign patients ^(45), (46), (47), (48), (49)^. However, one study that included patients from abroad for treatment reported no outstanding expenses ^(50)^.

**Table 2-c.** Overview of Single-Center, Retrospective Studies Presenting Unpaid Medical Benefits by Foreign Patients.

#	Author	Year	Duration	Study type	Location	Population	Total patients/facilities	Total unpaid medical expenses and cases	Key findings	Discussions on unpaid medical bills
11	Osegawa, et al. ^(45)^	2002	1993-2000	Retrospective review of medical records, Single Center	Chiba	Foreign patients visiting the emergency department	82	20%-30% (estimate)	24 patients did not speak English, which made obtaining informed consent almost impossible. The severity of foreign patients was higher than average. Most patients requested early discharge, which required many human resources and effort.	Repaying high medical bills takes long years and is challenging to collect. Visiting friends and relatives (VFR) from low- and middle-income countries without travel insurance and people accused of crimes were also included. Japanese legislation prohibits physicians from not providing patients with medical support; thus, financial support is of great necessity.
12	Ishikawa et al. ^(46)^	2003	1994-2000	Retrospective review of medical records, Single Center	Tokyo	Foreign patients admitted to a tertiary care center	168	Approximately 20% of bills have been unpaid over the last 7 years.	Patients were mainly from nearby countries (Korea, China, etc.) Leading causes of hospitalization were extrinsic factors such as violence and injury (male) and drug poisoning (female).	The reimbursement by the Traveler’s Law covered 5 patients (100%), while the Foreigner Unpaid Medical Cost Reimbursement system covered only 70%. Most local governments do not have a reimbursement system.
13	Kainuma et al. ^(47)^	2019	April 2014 to March 2019	Retrospective review of medical records, Single Center	Osaka	Foreign patients visiting the emergency department	198	364260 JPY, 6 cases	Communication was mainly possible with the usage of an interpreting device. Unpaid medical expenses became a problem, although many tourists had travel insurance.	Japanese hospitals cannot refuse emergency medical care from a humanitarian and ethical standpoint. Several public aids are available for medical facilities with unpaid medical bills by foreign patients. Legislative measures for infectious diseases and systems such as workers’ compensation insurance and mandatory vehicle liability insurance may be usable under specific circumstances. Assisting foreign patients to visit free/low-cost medical services may be an option. The hospital explained the medical expenses before consultations, but unpaid medical expenses occurred with patients who were guaranteed to pay afterward but lost contact.
14	Suzaki et al. ^(48)^	2019	April to September 2017	Retrospective review of medical records, Single Center	Tokyo	Foreign patients visiting the primary and secondary emergency departments for the first time	158	3 cases (amount unspecified)	Infectious diseases, which were common diseases, were also common among foreign patients. Primary and secondary emergency departments also had a high number of injuries. Some cases included several refusals of other medical facilities. 8% of patients with residency in Japan did not have national healthcare insurance.	The three cases were all by residents without national health insurance. The financial burden on medical institutions with unpaid medical bills is significant, and backup from the government is essential.
15	Shimoyama et al. ^(49)^	2020	April 2015 to March 2018	Retrospective review of medical records, Single Center	Tokyo	Foreign patients transported to a tertiary care center	87	13 cases (6 partially unpaid, 7 fully unpaid), 13,126,561 JPY	42% of the patients were covered by Japanese public healthcare insurance. 54% of the cases required linguistic assistance, but professional interpreting was provided in only one case. Difficulty was found in gaining informed consent and planning international transfers.	Unpaid medical expenses occur more often with patients uncovered by insurance (23.9%) than insured patients (2.4%). The total unpaid bills were high, costing up to 10.1% of the medical bills by foreign patients. Only 0.13% of patients, including Japanese patients, have unpaid medical bills. Hence, the rate of unpaid medical bills among foreigners is high. Credit card usage during night-time is needed to prevent unpaid bills. Presenting estimated costs, promoting prepayment, and using payment guarantee companies are to be considered.
16	Shirozu et al. ^(50)^	2021	1997- August 2020	Retrospective review of medical records and questionnaire, Single Center	Tokyo	Patients with hypothalamic hamartoma who underwent stereotactic radiofrequency thermocoagulation	65 foreign patients (159 domestic patients as control)	None	Foreign patients were younger and underwent aggressive surgery, resulting in transient complications, but the final results were not different. The majority were satisfied with the treatment. Medical staff presented problems with communication, rule compliance, and meals.	Prepayment of medical bills resulted in no unpaid medical expenses.

The year of publication, duration and location of the study, study population, total number of patients or facilities included in the study, total amount of unpaid medical expenses reported, key findings, and discussions on unpaid medical bills were derived from each study. JPY, Japanese yen.

Studies in the emergency department surmised that patients without healthcare insurance, both travel and public health insurance, were at risk for unpaid medical expenses. One report successfully gained reimbursement from the government under Traveler’s Law ^(46)^. Nevertheless, most studies have noted that legislative measures to protect medical facilities from facing financial risk from the unpaid medical expenses of foreign patients are lacking ^(45), (46), (47), (48)^.

The study population, which did not experience any outstanding expenses, was not from the emergency department and utilized the coordination of patients by collecting payments before patient acceptance ^(50)^.

## Discussion

This scoping review synthesized previous research conducted in Japanese medical facilities that reported unpaid medical bills by foreign patients. To the authors’ knowledge, our study is the first to concentrate on outstanding expenses incurred by foreign patients despite the significance of this issue for Japanese healthcare institutions. If this problem remains unresolved, medical facilities may hesitate to provide care to foreign patients.

However, our study underscores the paucity of research investigating the current status and preventive measures against unpaid medical bills. Two themes emerged from the existing studies.

### Prevalence of unpaid medical bills

The first theme is the prevalence of unpaid medical bills. In most medical institutions, treating foreign patients has led to the prevalence of unpaid medical bills. All studies in this review covering foreign patients in emergency departments reported outstanding expenses ^(45), (46), (47), (48), (49)^. Foreign patients tend to visit emergency departments outside regular hours (i.e., at night or weekends) ^(16)^, which may lack the necessary resources to handle such patients, such as medical interpreters or international medical coordinators. The complexity of linguistic, cultural, and social barriers exacerbates this challenge. Conversely, instances where no unpaid bills were reported involved medical travel cases with prepayment ^(50)^. With respect to patients with residency status, both travelers and residents were at risk of outstanding expenses.

### Preventing outstanding expenses

Among the topics discussed, the authors were concerned mainly with preventing unpaid medical bills. Four primary strategies were noted: insurance coverage, proactive strategies, such as prepayment or presenting estimated costs beforehand, legislative measures, and public policies to support medical facilities financially.

Insurance coverage was critical concerning the patients’ status. Although residents in Japan are eligible for public health insurance, some foreign residents do not possess such coverage ^(40), (42), (48)^. Some were due to the expiration of their residency status, ^(35), (36), (37)^, but other studies have suggested other difficulties for foreigners in obtaining public healthcare insurance ^(8)^. These patients incur total medical expenses out-of-pocket and are ineligible for the maximum healthcare expenses capping system ^(28)^, leading to costly monthly payments ^(41)^. Some foreign residents in Japan may lose access to public insurance if they fail to pay public health insurance premiums, or if they are unaware of the process for transitioning from employer-sponsored to municipal insurance ^(30)^. For example, technical interns who leave their sponsoring companies may become uninsured unless they proactively enroll in National Health Insurance and continue premium payments. In addition, Japan’s income-based cap on medical expenditures is only available to those who declare income through official channels ^(30)^, which may be difficult for migrants. These systemic issues may disproportionately expose immigrants to financial hardship and unpaid medical expenses, even if they reside in Japan for extended periods. Language barriers affect healthcare outcomes ^(52), (53)^, but financial barriers can also negatively affect the quality of healthcare received.

Foreign visitors are highly encouraged to enroll in travel insurance, as travelers without such coverage were reported to have unpaid medical bills. A characteristic of foreign tourists not purchasing travel insurance, as suggested by the study, was visiting friends or relatives (VFR tourists) in Japan ^(45)^. VFR tourists are known to be vulnerable in healthcare due to various risks, including low insurance coverage and disease risk misperception ^(54)^. This trend is similar among VFR tourists in Japan. A survey by the Japan Tourism Agency reported that approximately 73% of foreign tourists visiting Japan purchased travel insurance, a rate that has remained unchanged over the past six years, despite efforts to increase it ^(55)^. However, travel insurance itself does not guarantee payment, as it may require discussions with the insurance company regarding its coverage policy. Indeed, one study reported unpaid medical bills despite having traveler insurance ^(47)^.

Proactive strategies, such as prepayment and presenting estimated costs in advance have been proposed ^(36), (38), (39), (49)^. One study successfully did not experience outstanding expenses by collecting payments before patient acceptance ^(50)^. Presenting estimated costs and prepayment may be helpful, particularly for patients traveling to seek medical care, as was the case in this case ^(50)^. However, this approach requires resources, such as an international medical coordinator to maintain contact with the patient and medical administrators capable of calculating estimated costs. A banking system that accepts international transactions or an online credit card payment system would also be necessary for prepayment requests. Nonetheless, one study reported unpaid medical expenses despite presenting medical costs beforehand ^(47)^. Therefore, other preventive methods are essential. Moreover, presenting estimated medical costs before treatment is challenging in emergency rooms, as determining the necessary treatment without proper analysis is difficult. As unpaid medical expenses are particularly concerning in emergency departments, other comprehensive strategies are needed.

Studies have also noted several legislative measures that can aid the payments of foreign patients. One study reported a successful claim under Traveler’s law ^(46)^. The Traveler’s law (*Kōryo Byōnin oyobi Kōryo Shibōnin Toriatsukai-hō*) provides a framework for managing individuals who fall ill or die while traveling, especially when their identity or place of residence is unknown ^(56)^. Although payment is billed to the family when it is found, the local government is responsible for paying medical bills under this law. However, applying this law has proven challenging ^(36)^, making this system difficult to rely upon. Other legislative measures are also available. For example, medical costs for specific infectious diseases, such as tuberculosis are covered by public funding ^(57)^. If the patient is a resident, those receiving social welfare have medical costs covered by public funds, although only a limited number of patients qualify for these programs.

Finally, some public sector support systems are available ^(36), (39), (42), (44), (46), (48)^. Certain prefectures offer subsidies for unpaid medical expenses by foreign patients in emergency departments. Tokyo is one such prefecture; although its policies cap the maximum amount of subsidies per case, government-operated hospitals cannot apply for such funding ^(58)^. Similarly, some local governments provided subsidies for emergency centers with unpaid medical bills. Yamanashi Prefecture is one example, but the limit is up to 70% of the original outstanding expenses ^(59)^. These measures may alleviate the financial burden on healthcare facilities, but such governments are limited, and the measures are insufficient to prevent outstanding expenses.

Despite these measures, outstanding expenses remain unresolved, underscoring the need for comprehensive strategies. Therefore, developing effective interventions to address unpaid medical bills among foreign patients in Japan is crucial to ensure a better healthcare experience for all.

### Implications for future studies

Despite the growing demand and interest in studies on unpaid medical expenses by foreign patients, few studies have explicitly focused on this topic, necessitating further research. This review unraveled potential research questions.

First, a comparison with Japanese patients is needed. Few studies have compared the number of cases or the total amount of outstanding expenses with Japanese patients ^(37), (49)^, limiting the scientific validity of the hypothesis that exceptional expenses by foreign patients are more concerning than financial burdens from Japanese patients. Such studies are necessary to understand this agenda’s current situation more clearly, especially to identify the culprits of this agenda, such as healthcare and travel insurance coverage and adequate welfare service access for residents.

Second, information specifically for the coverage of healthcare insurance should be gathered. Although the studies included in this review called for the importance of healthcare insurance, many studies did not specify the type of insurance of the patients. This may be difficult to obtain retrospectively, especially for travelers, as patients may try to request coverage after paying upfront and returning to their homeland. As several studies noted that foreign residents may also not be enrolled in the Japanese Public Healthcare Insurance system, the enrollment of foreign residents is also an essential topic for investigators.

Third, more studies should evaluate potential prevention strategies from the perspectives of healthcare facilities and policymakers. Multiple payment methods, such as credit cards, intervention by healthcare interpreters, and other necessary coordination, may prevent outstanding expenses, but these measures have not been validated. Furthermore, in addition to the Japan Tourism Agency’s efforts to promote travel insurance purchases ^(55)^, the Ministry of Health, Labour and Welfare has also implemented systems for medical facilities to report overseas patients with unpaid medical bills to immigrate ^(60)^ to prevent outstanding expenses. The effectiveness of these policies should also be examined and adequately refined if necessary.

### Limitations

This scoping review has several limitations that require attention. First, publication bias must be acknowledged despite conducting a comprehensive search, including multiple English and Japanese databases. Case reports tend to focus on clinical pearls rather than contents requiring efforts from social backgrounds. A previous study noted the possibility of Japanese medical facilities accepting foreign patients not routinely recording these experiences, decreasing the number of studies ^(51)^. Second, generalizability should be considered. Some studies included in this review were published more than 20 years ago. While this provides an idea of the context, the number of foreign residents and tourists and policies and legislative measures have changed over time. Furthermore, most studies have been conducted in urban areas, warranting attention to other regions of Japan.

### Conclusion

This review provides an overview of previous studies conducted in Japanese medical facilities that reported unpaid medical bills by foreign patients. The issue of unpaid medical bills for foreign patients is a widespread concern among various healthcare institutions nationwide. These institutions are implementing innovative communication strategies with patients to mitigate this problem. Although specific legal frameworks and public systems offer potential solutions to cover unpaid bills, relying solely on unpaid bills is challenging.

While there has been incremental progress in studies addressing unpaid medical bills, further measures and investigations are essential. Additional research is necessary to develop effective interventions and improve the overall healthcare experience for foreign patients.

## Article Information

### Acknowledgments

The author thanks his colleagues for helpful discussions on this topic, especially the Clinical Researcher Development Committee of National Center for Global Health and Medicine. The author acknowledges the use of Curie (American Journal Experts, LLC., North Carolina, USA) and Grammarly (Grammarly Inc, San Fransico, USA) for primary language editing. The views expressed in this manuscript are those of the author and do not necessarily represent the author’s institutions. The abstract of this manuscript was presented in the Joint Congress on Global Health 2024 (65th Annual Meeting of JSTM, 39th Annual Meeting of JAGH and 1st Annual Meeting of TAGHI) on November 16 and 17, 2024.

### Author Contributions

Soichiro Saeki conceptualized and prepared the methodology of the project. Soichiro Saeki conducted the investigation and formal analysis, and Hatsune Kido validated it. Eri Yamada, Yan Zhang, Kaori Minamitani, and Ryo Kawasaki provided insights into the visualization process. Soichiro Saeki prepared the original draft, and Hatsune Kido, Eri Yamada, Yan Zhang, Kaori Minamitani, Chihaya Hinohara, and Ryo Kawasaki reviewed and edited the manuscript. Kaori Minamitani, Chihaya Hinohara, and Ryo Kawasaki supervised the study. All authors read and approved the final manuscript.

### Conflicts of Interest

None

### IRB Approval Code and Name of the Institution

Not applicable

## Supplement

Supplementary Material
