# Supplementary material for: Unpaid Medical Expenses by Foreign Patients in Japan: A Scoping Review
Source: JMA J. 2025 Dec 26;9(1):10–23. doi: 10.31662/jmaj.2024-0418 (PMC12889202; doi:10.31662/jmaj.2024-0418)
Supplement: Supplementary Material [file 2433-3298-9-1-0010-s001.pdf]

Supplementary Material

S1. List of Gray Literature included in the study (in English).

Conference Proceedings / Abstracts

1. Takashina K, et al. [Issues in medical care for foreign visitors to Japan (especially concerning unpaid medical bills)]. Nippon Sekijuji Igaku. 2019;71(1):197.
2. Sato N, Suga H, Nakajima M. [Initiatives to reduce unpaid medical bills]. Nippon Sekijuji Igaku. 2016;68(1):132.
3. Ishikawa Y, Someya K. [Efforts to collect unpaid medical bills]. Nippon Sekijuji Igaku. 2016;68(1):132.
4. Arakawa Y, et al. [Responding to the rapidly increasing number of foreign travelers in inpatient care: Initiatives regarding medical fee payment methods]. Nippon Sekijuji Igaku. 2016;68(1):132.
5. Nagai H, Watanabe T. [Reconstruction of unpaid bill management and efforts to refuse medical care to malicious non-paying patients]. Nippon Sekijuji Igaku. 2023;73(1):244.
6. Hirano M, et al. [System construction and challenges of a support team for foreign patients]. Nippon Sekijuji Igaku. 2023;73(1):238.
7. Mori A. [Efforts to reduce assessment and return deductions]. Nippon Sekijuji Igaku. 2016;68(1):132.
8. Shimada R. [Status of unpaid medical bills by foreign patients in Kyoto Prefecture based on a Ministry of Health, Labour and Welfare survey]. Nihon Kango Kenkyu Gakkai Zasshi. 2003;26(3):3_443.
9. Arakawa Y, et al. [Initiatives for responding to the hospitalization of a rapidly increasing number of foreign travelers - Medical fee payment methods]. Takayama Sekijuji Byoin Kiyo. 2017;40:84.
10. Kon T. [Hospital management forum: Unpaid medical bills for foreign patients: Calling for the establishment of "social resources" for foreign patients]. Byoin. 1995;54(6):612-3.
11. Matsuo M. [Measures for unpaid medical bills of foreign patients at Toyota Memorial Hospital]. Byoin. 1995;54(5):500-1.
12. Sato H, Yokota H, Kushimoto S, Kawai M, Araki H, Atsumi I, Yokobori M, Fuse A, Fukuda R, Yamamoto Y. [Medical collaboration for socially vulnerable people: Economic issues in

medical care for socially vulnerable foreigners]. Journal of Japanese Association for Acute Medicine. 2008;11(2):123.
13. Du M, Futami A, Kamizato A. [Case report: Measures to prevent unpaid medical bills for foreign inpatient visitors to Japan]. Journal of the Japanese Society of Travel Medicine. 2018;12(Suppl.):89.
14. Hori N. [Tokyo Olympic and Paralympic Games: Explanatory materials for foreign patients, measures for medical bills (unpaid bills), and establishment of an external communication system]. Journal of Japanese Association for Acute Medicine. 2018;21(2):217.
15. Akashi M, Futami A, Nagai Y, Hori N. [Management of unpaid bill prevention in Chinese interpreter intervention cases]. Kokusai Hoken Iryo. 2018;33(3):171-2.
16. Futami A, Morishita K. [The role of the Japanese Association for Acute Medicine in Consortium 2020: From Tokyo 2020 to Osaka 2025: Establishment of a system for accepting inbound patients and risk assessment using unpaid bill triage]. Journal of Japanese Association for Acute Medicine. 2019;22(2):251.
17. Irimajiri S. [Medical institutions need to be prepared to handle difficult cases involving foreigners: Language barriers, cultural differences, and the problem of unpaid medical bills]. Journal of Medical English Education. 2020;19(2):11.
18. Katsuhara T, Otani N, Kajimoto R, Yoshino A. [A report on post-treatment management for undocumented individuals transported by emergency services]. Neurosurgical Emergency. 2024;28(3):3019.

Special Issues & Commentary

1. Taka S. [Foreign workers and challenges of the social security system]. Nippon Rodo Kenkyu Zasshi. 2022;64(7):55-65.
2. Takagi Y, Kato S, Yamashita Y. [Background of foreign patients: Issues to be considered besides medical treatment]. Gekkan Chiiki Igaku. 2020;34(3):56.
3. Ogasawara N, Aoki Y. [Development of internal medicine in an era of increasing foreign patients]. Nihon Naika Gakkai Zasshi. 2019;108(4):876-86.
4. Nakanishi I. [Better medical care for foreigners for primary care: What to do about medical bills: Efforts to prevent unpaid medical bills]. Chiryo. 2006;88(9):2285-8.
5. Kataoka Y. [Better medical care for foreigners for primary care: What to do about medical bills: System for medical institutions to claim from the government: A system for covering unpaid medical bills]. Chiryo. 2006;88(9):2281-4.

6. Futami A, Hori N. [Internationalized medical, health, and welfare: Inbound and outbound: Inbound: Domestic challenges in medical care for foreigners]. Shoni Naika. 2017;49(6):851-4.
7. Hori N. [From the front lines of accepting foreign patients: Medical care in a multicultural society, Nippon: Mechanisms and ingenuity to prevent unpaid bills]. Byoin. 2017;76(12):984-5.
8. Kobayashi M, Motegi H. [The "already happened future" facing hospitals: The ideal way of providing medical care to foreign residents]. Byoin. 2018;77(4):310-5.
9. Hori N. [Essence of responding to foreign patients: Points for practical response to foreign patients: Key points for responding to foreign patients to avoid trouble]. Yakkyoku. 2018;69(7):2582-6.
10. Hori N. [From the front lines of accepting foreign patients: Medical care in a multicultural society, Nippon: Who should bear the unpaid bills of foreign visitors?]. Byoin. 2018;77(7):594-5.
11. Kutsumi K. [Diversity management: Responding to diversity: Case study: Kucchan Kosei Hospital: Responding to foreign patients visiting Japan]. Byoin. 2018;77(8):638-41.
12. Yamada H, Tamura J. [Travel medicine course for general clinicians: Medical care for foreign patients: What to do if a foreign patient visits the outpatient clinic: Measures for foreign patients' medical bills]. Shindan to Chiryo. 2018;106(11):1385-9.
13. Sakashita J. [Hospitals facing a multinational society: The reality of accepting patients in areas with a large number of foreign residents]. Byoin. 2019;78(7):503-7.
14. Hori N. [Medical care for foreigners for practitioners: Medical care for foreigners and future challenges: Minimizing the burden and risk on the front lines]. Chiryo. 2019;101(8):980-3.
15. Futami A, Ohara T, Koga H. [Tokyo 2020: What can orthopedic surgeons do?: Establishment of a system for accepting foreign patients for the Tokyo Olympics at hospitals and clinics: Various problems and countermeasures regarding medical care for foreign patients]. Seikei Saigai Geka. 2019;62(10):1239-46.
16. Futami A, Okawa J. [Preparing for the increasing international medical care: Challenges and countermeasures regarding payment for international medical care]. Shonika Rinsho. 2019;72(10):1523-7.
17. Yamada H. [The turning point in care for foreign pregnant women: "Challenges to overcome" to accept residents and tourists: Countermeasures for medical settings from data]. Josan Zasshi. 2020;74(2):90-8.

18. Futami A. [Tokyo 2020 Olympics are held! "Preparation" and "response" to foreign patients: Hospital case study: Responding to the increasing number of foreign patients toward the Tokyo 2020 Games]. Nursing BUSINESS. 2020;14(1):36-40.
19. Takagi Y, Kato S, Yamashita Y. [Background of foreign patients: Issues to be considered besides medical treatment]. Chiiki Igaku. 2020;34(3):222-6.
20. Hori N. [Q&A on solving problems in medical care for foreign patients in a multicultural society, Nippon: Problems with payment specific to foreign patients]. Byoin. 2020;79(4):318-9.
21. Futami A. [Various travel medicine-related issues that gynecologists and obstetricians should know about: Toward the Tokyo Olympics and Paralympics: The problem of unpaid bills for foreign patients]. Sanka to Fujinka. 2020;87(4):399-405.
22. Hori N. [Various travel medicine-related issues that gynecologists and obstetricians should know about: Toward the Tokyo Olympics and Paralympics: Points to note when responding to foreign visitors]. Sanka to Fujinka. 2020;87(4):376-80.
23. Ikeda N, Tamura J, Ishii D, Iwamura M, Yoshida K. [Medical team for resident and visiting foreigners]. Nihon Rinsho Jinshoku Gakkai Zasshi. 2020;8(1):26-32.
24. Norizuki S. [Update on emerging and re-emerging infectious diseases: Infectious diseases in the globalized era: The current status and challenges of the medical system for foreign visitors in the global era]. Nippon Rinsho. 2021;79(2):281-5.
25. Suzuki K, Morishita K, Futami A. [Emergency surgery for foreign patients: Case study: Responding to foreign patients not enrolled in health insurance: What you should know about unpaid bill countermeasures, social security, and communication methods]. LiSA. 2021;28(5):510-4.
26. Futami A. [Emergency surgery for foreign patients: Establishment of a system for medical institutions to accept foreign patients: What to check at the reception and about payment]. LiSA. 2021;28(5):490-4.
27. Sakamoto M. [Supporting foreign children: From an SDG perspective: From the front lines of medical care for foreign children: Current status and challenges]. Child Health. 2024;27(12):940-3.
28. Sawada T, Yasukawa K. [To ensure that people who have difficulty accessing insured medical care are not left behind: A challenge in "immigrant health" in the SDG era: The current state of emergency medical care for uninsured foreigners and the need for a systematic response]. Kokusai Hoken Iryo (Journal of International Health). 2024;39(2):37-9.

Case Reports

1. Fujioka N, Nonomura K. [About unpaid bill collection work in the medical affairs department]. Kyoto Daini Sekijuji Byoin Igaku Zasshi. 2015;36:99-101.

Dissertations / Academic Bulletins

1. Okubo T. [National Health Insurance System and Overstaying Foreigners]. Waseda University; 2004.
2. Wu H. [Acceptance of foreign patients and medical tourism in medical institutions]. Osaka Keizai Hoka Daigaku Keizai-gaku Ronshu. 2019;43(1):1-18.
3. Ueki R, Okawa Y. [Current status and challenges of medical institutions in Fukui Prefecture regarding the acceptance of foreign patients]. Fukui Kenritsu Daigaku Ronshu. 2021;55:141-53.
4. Numao N. [Increase in foreign residents and challenges for local government administration and finance]. Kenkyujo Shiryo. 2021;133(1):105-17.

MHLW & Policy Reports

1. Endo H, Okamura S, Toya M. [Research on the development of medical institutions for accepting foreign patients]. Kosei Rodo Kagaku Kenkyuhi Hojokin Chiiki Iryo Kiban Kaihatsu Suishin Kenkyu Jigyo Sokatsu-Buntan Kenkyu Hokokusho.
2. Kitagawa Y, Yagi H, Okamura S. [Research for understanding the reality of unpaid bills from foreign patients visiting Japan and considering prevention measures in emergency medical institutions]. Kosei Rodo Kagaku Tokubetsu Kenkyu.

S2. List of Gray Literature included in the study (original Japanese list).

Conference Proceedings / Abstracts

1. 高階謙一郎, et al.「訪日外国人医療における課題（特に未収金対応）.」日赤医学 71.1 (2019): 197.
2. 佐藤尚子, 菅裕明, 中島雅己.「未収金削減に向けての取り組み.」日赤医学 68.1 (2016): 132.
3. 石川佳彦, 染谷恵子.「医業未収金回収の取り組みについて.」日赤医学 68.1 (2016): 132.
4. 荒川幸雄, et al.「急増する外国人旅行者の入院時対応の取り組み～医療費の支払方法について～.」日赤医学 68.1 (2016): 132.
5. 長井英行, 渡邉孝子.「未収金管理の再構築と悪質な未収患者に対する診療拒否への取り組み.」日赤医学 73.1 (2023): 244.
6. 平野美樹子, et al.「外国人患者対応サポートチームのシステム構築とその課題.」日赤医学 73.1 (2023): 238.
7. 森敦志.「査定・返戻の減額への取り組み.」日赤医学 68.1 (2016): 132.
8. 嶌田理佳.「厚生労働省調査にみる京都府内の外国人患者による医療費未払いの現状.」日本看護研究学会雑誌 26.3 (2003): 3_443.
9. 荒川幸雄, et al.「急増する外国人旅行者の入院時対応の取り組み～医療費の支払方法について～.」高山赤十字病院紀要 40 (2017): 84.
10. 洪東基.「病院管理フォーラム 外国人医療費の未収金 外国人患者の「社会資源」の確立を求めて」. 病院, vol. 54, no. 6, 1995, pp. 612-613
11. 松尾雅次.「トヨタ記念病院における外国人医療費の未収金対策」. 病院, vol. 54, no. 5, 1995, pp. 500-501
12. 佐藤秀貴, 横田裕行, 久志本成樹, 川井真, 荒木尚, 渥美生弘, 横堀将司, 布施明, 福田令雄, 山本保博.「社会的弱者に対する医療連携 社会的弱者の外国人医療に於ける経済的問題点」. 日本臨床救急医学会雑誌, vol. 11, no. 2, 2008, pp. 123.
13. 杜曼君, 二見茜, 上里彰仁.「訪日外国人入院患者の未収金防止対策 事例報告」. 日本渡航医学会誌, vol. 12, Suppl., 2018, pp. 89.
14. 堀成美.「東京オリンピック・パラリンピック 外国人患者への説明資料、医療費(未収金)対策、院外連絡体制の整備」. 日本臨床救急医学会雑誌, vol. 21, no. 2, 2018, pp. 217.
15. 明石雅子, 二見茜, 永井佑佳, 堀成美.「中国語通訳介入事例の未収金防止のマネジメント」. 国際保健医療, vol. 33, no. 3, 2018, pp. 171-172.

16. 二見茜, 森下幸治.「コンソーシアム 2020 における臨床救急医学会の役割～東京 2020 から大阪 2025 へ～ インバウンド患者受け入れ体制の整備 未収金トリアージを用いたリスクアセスメント」. 日本臨床救急医学会雑誌, vol. 22, no. 2, 2019, pp. 251.
17. 入交重雄.「医療機関は外国人困難事例への対応準備が必要である 言語障壁、文化相違、医療費未払い問題など」. Journal of Medical English Education, vol. 19, no. 2, 2020, pp. 11.
18. 勝原隆道, 大谷直樹, 梶本隆太, 吉野篤緒.「救急搬送された不法滞在者に対する治療後対応に関する報告」. Neurosurgical Emergency, vol. 28, no. 3, 2024, pp. 3019.

Special Issues & Commentary

1. 嵩さやか.「外国人労働者と社会保障制度の課題.」日本労働研究雑誌 64.7 (2022): 55–65.
2. 高木陽一, 加藤宗一郎, 山下ゆかり.「外国人患者の背景—医学的対処以外で配慮すべき問題点—.」月刊地域医学 34.3 (2020): 56.
3. 小笠原功明, 青木康博.「外国人患者増加時代における内科医療の展開.」日本内科学会雑誌 108.4 (2019): 876–886.
4. 中西泉. 「【プライマリ・ケアのためのよりよい外国人診療】医療費をどうするか 医療費の未収を生まない努力」. 治療, vol. 88, no. 9, 2006, pp. 2285-2288.
5. 片岡優子. 「【プライマリ・ケアのためのよりよい外国人診療】医療費をどうするか 医療機関が行政に請求できる制度 未収医療費補填制度」. 治療, vol. 88, no. 9, 2006, pp. 2281-2284.
6. 二見茜, 堀成美. 「【国際化する医療・保健・福祉-インバウンド・アウトバウンド】インバウンド 外国人医療における日本国内の課題」. 小児内科, vol. 49, no. 6, 2017, pp. 851-854.
7. 堀成美. 「外国人患者受け入れの現場から 多文化社会 NIPPON の医療 未収金を発生させない仕組み・工夫」. 病院, vol. 76, no. 12, 2017, pp. 984-985.
8. 小林幹男, 茂木寛. 「【病院が直面する「すでに起こった未来」】外国人居住者に対する医療提供のあり方」. 病院, vol. 77, no. 4, 2018, pp. 310-315.
9. 堀成美. 「【訪日外国人数増加と輸入感染症 外国人患者対応のエッセンス】外国人患者対応の実践ポイント トラブルを回避するための外国人患者対応の勘所」. 薬局, vol. 69, no. 7, 2018, pp. 2582-2586.

10. 堀成美. 「外国人患者受け入れの現場から 多文化社会 NIPPON の医療 訪日客の未収金は誰が負担すべきか」. 病院, vol. 77, no. 7, 2018, pp. 594-595.
11. 九津見圭司. 「【ダイバーシティ・マネジメント-多様性に対応する】事例 倶知安厚生病院 訪日外国人患者への対応」. 病院, vol. 77, no. 8, 2018, pp. 638-641.
12. 山田秀臣, 田村純人. 「【一般臨床医のための渡航医学講座】外国人患者の診療 外国人の患者が外来を受診したら 外国人患者の医療費対策」. 診断と治療, vol. 106, no. 11, 2018, pp. 1385-1389.
13. 坂下重吾. 「【多国籍社会に直面する病院】在留外国人が多い地域での患者受け入れの実際」. 病院, vol. 78, no. 7, 2019, pp. 503-507.
14. 堀成美. 「【実地医家のための外国人診療】外国人診療と今後の課題 現場の負担とリスクを最小限にするために」. 治療, vol. 101, no. 8, 2019, pp. 980-983.
15. 二見茜, 大原敏之, 古賀英之. 「【東京 2020:整形外科医には何ができるか?】病院・診療所における東京オリンピックに向けた外国人患者受入れ体制の整備 外国人患者診療に関する諸問題とその対応」. 整形・災害外科, vol. 62, no. 10, 2019, pp. 1239-1246.
16. 二見茜, 大川淳. 「【増加する国際診療に備える】国際診療の支払いに関する課題と対応策」. 小児科臨床, vol. 72, no. 10, 2019, pp. 1523-1527.
17. 山田秀臣. 「【外国人妊産婦のケア転換期 在住者・旅行者を受け入れるための"乗り越え課題"とは】データから考える医療現場の対応策」. 助産雑誌, vol. 74, no. 2, 2020, pp. 90-98.
18. 二見茜. 「【2020 年、東京五輪開催!外国人患者への「備え」と「対応」】病院事例 増加する外国人患者への対応 東京 2020 大会に向けて」. Nursing BUSINESS, vol. 14, no. 1, 2020, pp. 36-40.
19. 高木陽一, 加藤宗一郎, 山下ゆかり. 「外国人患者の背景 医学的対処以外で配慮すべき問題点」. 地域医学, vol. 34, no. 3, 2020, pp. 222-226.
20. 堀成美. 「多文化社会 NIPPON の医療 外国人患者受け入れの課題解決 Q&A 外国人患者特有の支払いの問題」. 病院, vol. 79, no. 4, 2020, pp. 318-319.
21. 二見茜. 「【産婦人科医も知っておきたい旅行医学関連の諸問題～東京オリンピック・パラリンピックに向けて】外国人患者の未収金問題」. 産科と婦人科, vol. 87, no. 4, 2020, pp. 399-405.

22. 堀成美. 「【産婦人科医も知っておきたい旅行医学関連の諸問題～東京オリンピック・パラリンピックに向けて】外国人受診者の対応上の注意点」. 産科と婦人科, vol. 87, no. 4, 2020, pp. 376-380.
23. 池田成江, 田村純人, 石井大輔, 岩村正嗣, 吉田一成. 「在留・訪日外国人対応の医療チーム」. 日本臨床腎移植学会雑誌, vol. 8, no. 1, 2020, pp. 26-32.
24. 法月正太郎. 「【新興・再興感染症 update-グローバル化時代の感染症-】グローバル時代の訪日外国人への医療制度の現状と課題」. 日本臨床, vol. 79, no. 2, 2021, pp. 281-285.
25. 鈴木啓介, 森下幸治, 二見茜. 「【外国人患者への緊急手術】ケーススタディ 健康保険に加入していない外国人患者への対応 知っておきたい未収金対策と社会保障,コミュニケーション方法」. LiSA, vol. 28, no. 5, 2021, pp. 510-514.
26. 二見茜. 「【外国人患者への緊急手術】医療機関の外国人患者受入れ体制整備 受付時に確認することと支払いについて」. LiSA, vol. 28, no. 5, 2021, pp. 490-49432.
27. 坂本昌彦. 「【外国人の子どもを支援する～SDGs の視点から～】外国人の子どもの診療現場から 現状と課題」. チャイルド ヘルス, vol. 27, no. 12, 2024, pp. 940-943.
28. 沢田貴志, 安川康介. 「保険診療へのアクセスが困難な人たちが取り残されないために-SDGs 時代の「移民の健康」における 1 課題 無保険外国人の緊急医療の現状と制度的対応の必要性」. 国際保健医療, vol. 39, no. 2, 2024, pp. 37-39.

Case Reports

1. 藤岡直大, 野々村公子. 「医事課における未収金回収業務について.」京都第二赤十字病院医学雑誌 36 (2015): 99–101.

Dissertations / Academic Bulletins

1.大窪高志.『国民健康保険制度と超過滞在外国人.』早稲田大学, 2004.
2. 呉紅敏, ゴコウビン.「医療機関における外国人患者の受け入れと医療ツーリズム.」大阪経済法科大学経済学論集 43.1 (2019): 1–18.
3. 上木礼子, 大川洋子.「福井県における外国人患者受け入れに対する医療機関の現状と課題.」福井県立大学論集 55 (2021): 141–153.

4. 沼尾波子.「在留外国人の増加と自治体行財政の課題.」研究所資料 133.1 (2021): 105–117.

MHLW & Policy Reports

1. 遠藤弘良, 岡村世里奈, 遠矢雅史.「外国人患者の受入れに関する医療機関の整備に関する研究.」厚生労働科学研究費補助金 地域医療基盤開発推進研究事業 総括・分担研究報告書.
2. 北川雄光, 八木洋, 岡村世里奈.「救急医療機関における訪日外国人患者の未収金の実態把握と防止策検討のための研究.」厚生労働科学特別研究.
